# Gastrointestinal Symptoms in Association With Hypokalemia Can Be a Predictor of Inferior Outcomes in COVID-19

**DOI:** 10.7759/cureus.14466

**Published:** 2021-04-13

**Authors:** Nicholas wong wai cheong, Veeraraghavan Meyyur Aravamudan, Jonathen Venkatachalam, Navin Kuthiah

**Affiliations:** 1 Department of Internal Medicine, Woodlands Health Campus, Singapore, SGP; 2 Department of Medicine, Woodlands Health Campus, Singapore, SGP; 3 Department of Respiratory Medicine, Khoo Teck Puat Hospital, Singapore, SGP

**Keywords:** covid 19, hypokalemia, gastrointestinal losses

## Abstract

The coronavirus disease 2019 (COVID-19) pandemic, caused by severe acute respiratory syndrome coronavirus disease-2 (SARS-C0V-2), has affected many lives globally. In Singapore, majority of the infected individuals are foreign workers residing in dormitories. A retrospective review conducted over two weeks (April 13 to April 26, 2020) of migrant workers admitted to a public hospital in Singapore revealed that a significant number of them developed hypokalemia. The purpose of this study was to examine any association that might exist between COVID-19 and hypokalemia. Fifty patients in this study had hypokalemia, translating to a prevalence of 28.4% (95% CI: 21.9-35.7). Gastrointestinal (GI) loss was a significant cause of hypokalemia with a prevalence of GI symptoms in the study group (diarrhea, vomiting, poor oral intake) of 5.7% (95% CI: 2.8-10.2). Clinicians should consider screening for hypokalemia in COVID-19 patients and initiate potassium replacement to mitigate any potential arrhythmias.

## Introduction

The coronavirus disease 2019 (COVID-19) was declared a pandemic by the World Health Organization (WHO) on March 11, 2020. To date, Singapore has seen several waves of COVID-19 infections since the outbreak began in Wuhan in late December 2019. The most recent and consequential waves were reported among foreign workers residing in dormitories. This constituted the majority of COVID-19 cases documented in Singapore. Fortunately, these individuals are predominantly young healthy individuals.

The virus responsible for this disease, severe acute respiratory syndrome coronavirus 2 (SARS-C0V-2), infects individuals, resulting in a myriad of symptoms. A significant number of afflicted individuals developed hypokalemia. Hypokalemia is defined as a potassium level of less than 3.5 mmol/L. Mild hypokalemia constitutes of potassium level between 3 mmol/L and 3.5 mmol /L, whereas moderate-severe hypokalemia is defined as a potassium level of less than 3.0 mmol/L.

This study describes the association between hypokalemia and COVID-19 infection in migrant workers admitted to two general medical wards in a public hospital in Singapore.

## Materials and methods

Methodology

Study Population

A total of 176 COVID-19 male patients admitted to two general medical wards in a public hospital in April 2020 were reviewed retrospectively. All these patients were migrant workers living in dormitories. Only 11 patients in this study group had preexisting medical conditions, of which three had hypertension while six others had diabetes mellitus. The remaining two patients had more than one cardiovascular risk factors, with one having diabetes and hypertension and the other having ischemic heart disease, hypertension, and dyslipidemia.

Table 1 depicts the baseline characteristics of our study population.

**Table 1:** Characteristics of patients included in the study

Characteristics	n
Male	176
Age (years)	
21-30	48
31-40	80
41-50	44
51-60	4
Diabetes mellitus	7
Hypertension	6
Hyperlipidemia	1
Ischemic heart disease	1

## Results

Study design

Relevant data pertaining to each patient on admission to the ward were anonymously extracted by an independent investigator using the ward census and inpatient clinical management software. All COVID-19 patients were diagnosed using real-time polymerase chain reaction (PCR). Data collected included age, potassium levels, need for potassium replacements, presence of gastrointestinal (GI) losses, poor oral intake, preexisting comorbidities, blood pressure range, and cycle threshold (CT) value of SARS-CoV-2. The blood pressure range throughout the inpatient stay was recorded. Isolated high or low blood pressure readings by more than 20/10 mm Hg were disregarded. The study was approved by the local Domain Specific Review Board (DSRB).

Statistical analysis

The data set collected in this study was analyzed using SPSS Statistics Version 25 (IBM Cop., Armonk, NY, USA) and RStudio Version 1.1.456 (RStudio, Boston, MA, USA) to ascertain the association between COVID-19 infection and hypokalemia. The prevalence of hypokalemia and GI symptoms in the study group was determined using the binomial test with Clopper-Pearson confidence interval (CI). The comparison between the two groups (hypokalemia and normokalemia) was examined using Fisher’s exact test. Independent Student’s t-test was used to analyze the correlation between potassium levels and CT values.

Results

**Figure 1:**
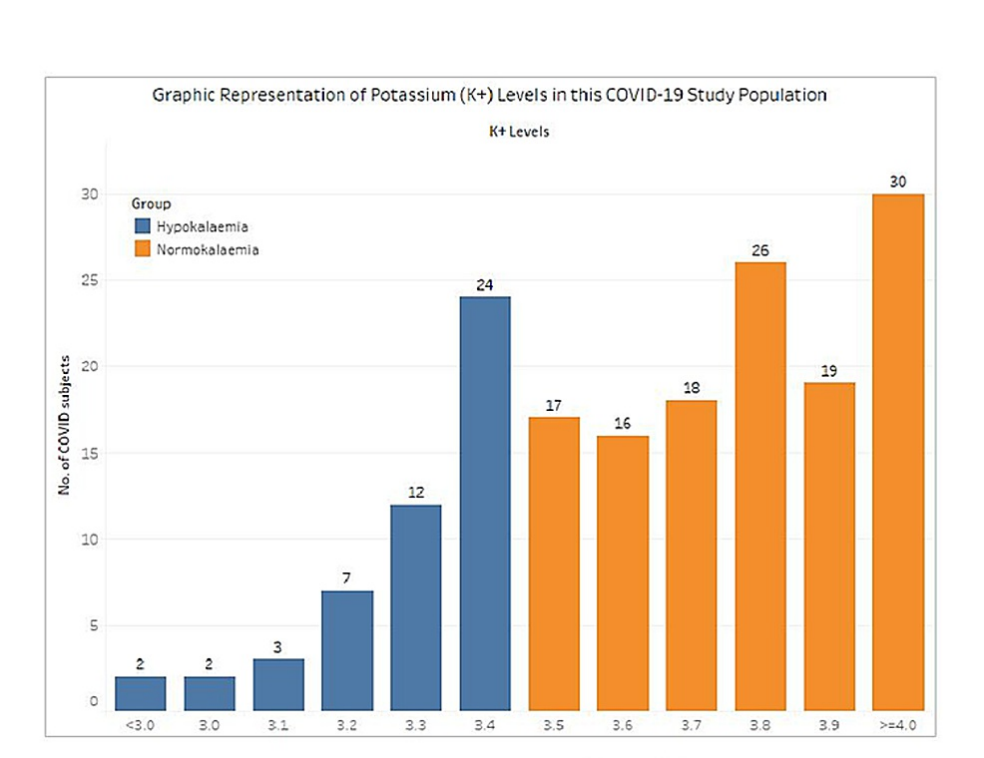
Graphic representation of serum potassium levels in COVID-19 population

In this cohort of 176 foreign migrant workers, the prevalence of hypokalemia was 28.4% (95% CI: 21.9-35.7%). Out of these 50 cases, 48 had only mild hypokalemia, whereas two had moderate hypokalemia. See Figure 1 for the breakdown of the study group based on potassium levels. None of the patients with hypokalemia had arrhythmias. Most patients were started on oral potassium supplementation and remained stable. One patient with mild hypokalemia of 3.4 mmol/L developed severe disease requiring invasive ventilation in the intensive care unit (ICU). All others remained stable and were managed in the general ward.

The prevalence of GI symptoms in the study group (diarrhea, vomiting, poor oral intake) was 5.7% (95% CI: 2.8-10.2%). When stratified according to the presence of GI symptoms, the prevalence of GI symptoms in the hypokalemic group was 12% (95% CI: 4.5%-24.3%) and that in the normokalemic group was 3.2% (95% CI: 0.9%-7.9%).

**Figure 2:**
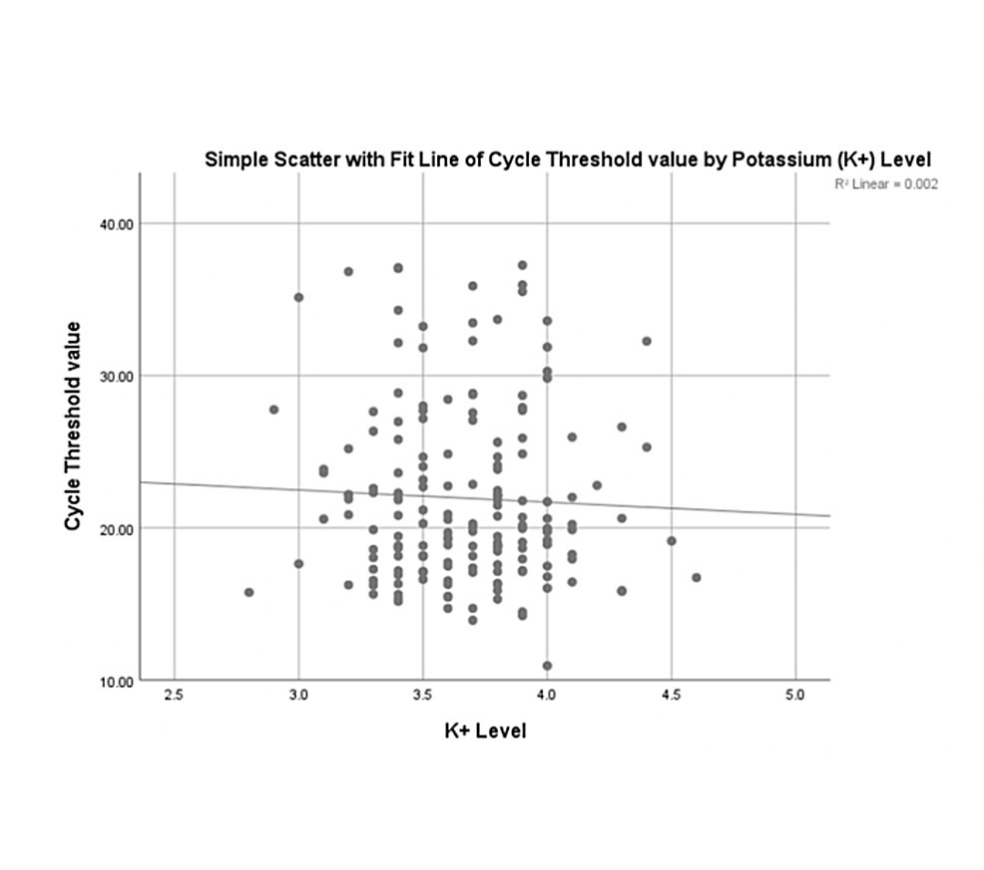
Simple scatter with fit line of cycle threshold value by potassium level

There is a statistically significant association between the potassium levels and GI symptoms (p=0.032), indicating that a higher proportion of hypokalemic patients had GI symptoms. The independent Student’s t-test demonstrated that there is no statistically significant difference in mean CT levels between the hypokalemic and normokalemic groups (mean for hypokalemic group = 22.4; mean for normokalemic group= 21.8; p=0.55). See Figure 2 for the simple scatter chart showing no linear relationship between CT levels and potassium levels. There are no clinical studies describing any association between CT value and hypokalemia, which can be useful in deciding which patients should have closer monitoring for hypokalemia.

**Figure 3:**
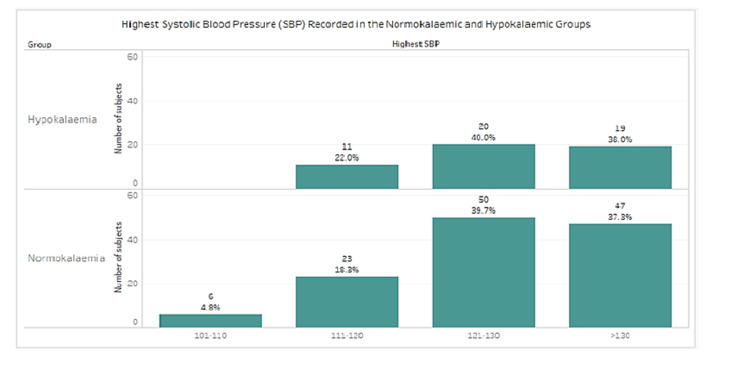
Highest blood pressure recorded in the normokalemic and hypokalemic groups

Highest systolic blood pressure (SBP) was recorded for all patients (Figure 3).

**Figure 4:**
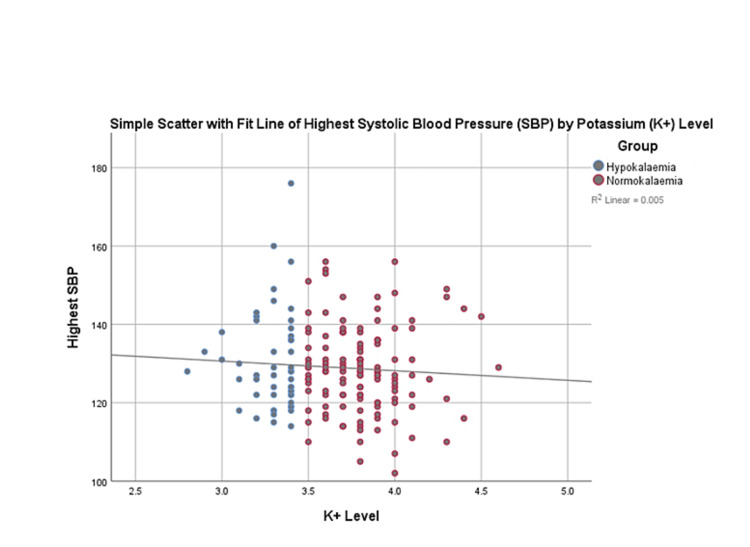
Simple scatter with fit line of highest systolic blood pressure and potassium levels

Using Fisher’s exact test, there is no significant association between the potassium level and the highest SBP range (p=0.5244) in this study population. See Figure 4, which demonstrates no linear relationship between the two variables.

## Discussion

Hypokalemia has been described as a sensitive biomarker of the severity of COVID-19, with hypokalemia being independently associated with invasive mechanical ventilation requirement [1]. In our study, 28.4% of patients admitted to the hospital with mild COVID-19 symptoms were found incidentally to have mild-to-moderate hypokalemia. This is much lower compared to a study of 175 COVID-19 patients by Chen et al., which showed a prevalence of 61.7% [2]. Hypokalemia has interestingly been described in other respiratory viral infections. A 2009 study by Cao et al. of patients with H1N1 infections showed that 25.4% of patients had hypokalemia [3]. Studies also showed that hypokalemia was found in 43% [4] to 90% [5] of patients with SARS.

The prevalence of hypokalemia in the general population is difficult to estimate. Studies indicate that 5.5% [6] to 39% [7,8] of people have hypokalemia. The studies involving inpatients and those presenting to the emergency departments show a higher prevalence of hypokalemia. There are no studies on the prevalence of hypokalemia in people not taking any medications, but it is estimated to be probably fewer than 1% [9].

While inpatients may have hypokalemia due to a gamut of reasons, all patients in our study were healthy young men who could potentially have been managed in the community. Most of them did not have any comorbidities and were not taking any medications or health supplements.

Although hypokalemia seems to be mild in most cases, it can result in adverse effects such as arrhythmias and muscle symptoms such as weakness and myalgia. Arrhythmias have been noted to occur in COVID-19 patients.

A study from the Hubei province stated that 7.3% of the patients presented with palpitations [10]. In another study by Wang et al., 17% of patients were found to have arrhythmias, with a higher number noted in the patients admitted to intensive care [11]. There are also studies describing atrial [12] and ventricular arrhythmias [13] in COVID-19 patients.

Given the possibility of hypokalemia triggering arrhythmias, it is imperative for potassium levels to be checked and appropriately managed. Also, the presence of hypokalemia may augment the arrhythmogenicity of hydroxychloroquine, which is a treatment consideration for COVID-19.

In our study, there was a positive correlation between hypokalemia and the presence of poor oral intake or GI losses. This is consistent with the study by Chen et al., which reported a 23% prevalence of GI losses in the study group [1]. However, the prevalence of GI losses in our study was much lower. GI symptoms are common in COVID-19. Pan et al. in a study of 204 patients found that 103 (50.5%) patients reported a digestive symptom, including lack of appetite (81 [78.6%] cases), diarrhea (35 [34%] cases), vomiting (4 [3.9%] cases), and abdominal pain (2 [1.9%] cases) [14].

Although GI losses certainly can precipitate hypokalemia, only 12% of patients with hypokalemia in our study had GI losses. This raises a possibility of another mechanism contributing to hypokalemia in COVID-19 patients is at play.

The ACE2 (angiotensin-converting enzyme 2) has been proven to be a cell receptor for SARS-CoV-2 from human and animal studies [15]. This is a similar mechanism implicated in the entry of SARS-CoV-2 into cells [16]. The downregulation of ACE2 leads to increase in angiotensin II, which can increase urinary losses of potassium. However, this is controversial as this theory has not been proven in any clinical or experimental studies. In addition, hypertension, which is a proxy of elevated angiotensin II levels, was not evident in our study.

We could not study the correlation of hypokalemia with disease severity as there was only one patient who needed intensive care in our study cohort. The patient with the lowest potassium level in our study (2.8 mmol/L) remained stable on intravenous and oral potassium supplementation.

There was no association between hypokalemia levels and CT values. At the moment, there are no clinical studies describing the association between CT value and hypokalemia. Our thinking was to determine if the CT value can be used as a predictor of hypokalemia, which will be then useful in deciding which patients should have a closer monitoring for hypokalemia.

One of the limitations of our study is that the results cannot be extrapolated to the general population as all the patients in our study were male migrant foreign workers. However, the relatively higher prevalence of hypokalemia in this cohort of healthy young men raises the possibility of an even higher prevalence in the general population. We were also not able to examine urinary losses amounting to hypokalemia in the study cohort because non-essential laboratory investigations were minimized in this pandemic situation. GI symptoms cause hypokalemia in all illnesses (viral/bacterial etc) and the same applies to COVID-19. Therefore, patients with GI symptoms in COVID-19 will obviously have some degree of hypokalemia. Given the low mortality rate of COVID-19 in Singapore, it is difficult to corroborate that hypokalemia may lead to increased adverse events.

GI loss is likely the reason for hypokalemia, but other possible mechanisms may play a role, such as poor oral intake. Correlation of hypokalemia with severity of illness such as ICU admission, need for ventilatory support, and need for usage of antiviral drug such as remdesevir was not possible due to the small sample size.

There are a few limitations of this study, such as small sample size and the involvement of only male foreign workers and healthy participants, which can contribute to limitation of the results.

It is a retrospective study and has its inherent limitations. It is a hypothesized generative study. Clinicians should be aware of the risks of hypokalemia in COVID-19 patients with GI symptoms and take actions early rather than late to reduce the hypokalemia-related complications, morbidity, and mortality in COVID-19 infection.

Certain COVID-19 patients may have increased risk of hypokalemia in the absence of renal disease or precipitating factors for hyperkalemia. This research is quite important in the COVID era in view of exploring the risk of hypokalemia and its related complications of arrhythmia in COVID-19 patients and raising the awareness of the clinicians in tackling this problem as early as possible.

While GI losses and other factors precipitating hypokalemia can be easily treated with potassium supplements, the study remains significant as it correlates with a potential risk of arrhythmias in patients.

## Conclusions

With the COVID-19 outbreak being long drawn and even possibly becoming endemic in certain parts of the world, many patients with mild illness will be managed in the community. As such, potassium levels should be monitored for COVID-19 patients. In the absence of any underlying risk factors of precipitating hyperkalemia (renal disease and any other possible causes of hyperkalemia), a low-dose potassium supplementation should be considered for all patients especially for those with poor oral intake and GI losses to mitigate the adverse effects of hypokalemia.
